# Supplementary material for: A Multidimensional Evaluation of the Factors in the Animal Welfare Assessment Grid (AWAG) That Are Associated with, and Predictive of, Behaviour Disorders in Dogs
Source: Animals (Basel). 2024 Feb 6;14(4):528. doi: 10.3390/ani14040528 (PMC10886356; doi:10.3390/ani14040528)
Supplement: Supplementary file 1 [file animals-14-00528-s001.zip › animals-2836276-supplementary.docx]

**Table S1**. Factor scores and their written descriptors.

| **Physical** |  |  |  |
| --- | --- | --- | --- |
| **Mobility** | **Body condition** | **Clinical assessment** | **Eating and drinking** |
| 1. the dog has very good mobility with no lameness or stiffness and is normally active or has normal energy | 1. ribs easily palpable without pressure, with minimal fat covering, waist easily noted and evident abdominal tuck | 1. clinically healthy, no injury or sign of disease | 1. eating and drinking as normal |
| 2. very good mobility with occasional mild stiffness and is normally active | 2. ribs fairly easy to palpate without pressure with thin fat covering and evident abdominal tuck from above | 2. mild transient subclinical symptoms or injury but has no evident behaviour change or impact on welfare | 2. food and / or water consumption is minimally reduced |
| 3. good mobility with short bouts of stiffness | 3. slight fat covering, slight pressure needed to palpate ribs, waist observable from above | 3. mild transient clinical symptoms or injury with mild transient behaviour change and impact on welfare | 3. mild to moderate reduced food / water (>20%) |
| 4. good mobility with generalised stiffness | 4. slight covering of fat, slight waist observable from above, can palpate ribs with pressure needed | 4. mild clinical symptoms or injury with mild behaviour change and impact on welfare | 4. moderately reduced food / water (>30%) |
| 5. moderate mobility, stiffness but frequently active | 5. moderate covering of fat, waist discerned from above but not prominent, can palpate ribs with pressure | 5. moderate transient clinical symptoms or injury with some behaviour change and impact on welfare | 5. moderately reduced food / water (>50%) |
| 6. moderate mobility, stiffness and less active | 6. excess covering of fat, no discernible waistline and difficulty palpating ribs | 6. moderate clinical symptoms or injury with moderate behaviour change and impact on welfare | 6. severely reduced food / water (>80%) |
| 7. poor mobility, stiffness and less active | 7. (overweight) heavy fat present and slight abdominal distension, difficult to palpate ribs or (underweight) ribs and shoulder visible with little fat | 7. moderate/severe disease or injury with moderate behaviour change and impact on welfare | 7. anorexic, has minimal loss of skin turgor |
| 8. very poor mobility, stiffness and minimally active | 8. (overweight) heavy fat present with abdominal distension, cannot palpate ribs or (underweight) ribs, lumbar vertebrae and pelvic bones somewhat visible with little detectable fat | 8. moderate/severe disease or injury with severe behaviour change and impact on welfare | 8. anorexic, has moderate loss of skin turgor, somewhat dry mucous membranes |
| 9. very poor mobility, stiffness and not at all active | 9. (overweight) very heavy fat present with obvious abdominal distension, cannot palpate ribs or (underweight) ribs, lumbar vertebrae and pelvic bones easily visible with very little fat | 9. severe disease and clinical symptoms or injury with severe of behaviour change and impact on welfare | 9. anorexic, has considerable loss of skin turgor, dry mucous membranes OR severe hunger / thirst |
| 10. Non-ambulatory and cannot move without assistance or support | 10. Massive fat deposits over neck thorax, spine, limbs and base of tail with obvious abdominal distention, cannot palpate ribs or ribs, lumbar vertebrae, pelvic bones and all bony prominences evident from a distance. No discernible body fat and obvious loss of muscle mass | 10. Extreme disease and clinical symptoms or injury with extreme behaviour change and impact on welfare | 10. Anorexic, has major loss of skin turgor, extremely dry mucous membranes OR severe and constant hunger / thirst |

| **Psychological** |  |  |  |
| --- | --- | --- | --- |
| **Aggression towards caregiver** | **Aggression towards unfamiliar people** | **Fears and anxieties frequency** | **Reaction to stressors** |
| 1. none | 1. none | 1. rarely encounters stressors | 1. displays minimal signs of fear and anxiety when encounters potential stressors |
| 2. occasionally growls, is predictable and trigger avoided | 2. occasionally growls, is predictable and trigger avoided | 2. encounters stressors a couple of times a year | 2. shows signs of fear to stressors and returns to normal <30 seconds |
| 3. occasionally growls, is predictable but trigger not always avoided | 3. occasionally growls, is predictable but trigger not always avoided | 3. encounters stressors multiple times a year | 3. shows signs of fear to stressors and returns to normal in minutes |
| 4. occasionally growls, is predictable but trigger rarely avoided | 4. occasionally growls, is predictable but trigger rarely avoided | 4. encounters stressors monthly | 4. shows signs of fear to stressors and some minor and returns to normal after 10 minutes |
| 5. occasionally snaps or bites, is predictable and trigger avoided | 5. occasionally snaps or bites, is predictable and trigger avoided | 5. encounters stressors weekly | 5. shows signs of fear to stressors and returns to normal after 30 minutes |
| 6. occasionally snaps or bites, is predictable but trigger not always avoided | 6. occasionally snaps or bites, is predictable but trigger not always avoided | 6. encounters stressors several times weekly | 6. shows signs of fear to stressors and takes up to an hour to return to normal |
| 7. occasionally snaps or bites, is predictable but trigger rarely avoided | 7. occasionally snaps or bites, is predictable but trigger rarely avoided | 7. encounters stressors daily | 7. shows signs of fear to stressors and takes several hours to return to normal |
| 8. bites, is somewhat predictable  and trigger largely avoided | 8. bites, is somewhat predictable and trigger largely avoided | 8. encounters stressors over 50% of the day | 8. shows signs of fear to stressors and takes most of the day to return to normal |
| 9. bites, is somewhat predictable and trigger not avoided | 9. bites, is somewhat predictable and trigger not avoided | 9. encounters stressors over 75% of the day | 9. shows signs of fear to stressors and takes several days to return to normal |
| 10. severe bites that are unpredictable | 10. severe bites that are unpredictable | 10. encounters constant stressors | 10. shows signs of fear to stressors and is always anxious |

| **Environment** |  |  |
| --- | --- | --- |
| **Choice, control, and predictability** | **Enrichment** | **Social** |
| 1. has good control over their environment and can make a range of choices, has highly predictable environment | 1. engaged in multiple forms of enrichment for over two hours daily | 1. has high-quality social interactions daily |
| 2. has good control over their environment and can make a range of choices, mostly has predictable environment | 2. engaged in multiple forms of enrichment for one to two hours daily | 2. has high-quality social interactions most days |
| 3. has some control over environment, can make some choices, has mostly predictable environment | 3. engaged in multiple forms of enrichment for up to one hour daily | 3. has good-quality social interactions daily |
| 4. has some control over environment, can make some choices, has some predictability | 4. engaged in enrichment for up to 30mins daily | 4. has good-quality interactions most days |
| 5. has little control over environment, can make some choices, has little predictability | 5. engaged with enrichment for less than 15mins daily | 5.  has good-quality interactions weekly |
| 6. spends several hours in an unpredictable environment, can make some choices | 6. somewhat engaged with enrichment several times weekly | 6.  has moderate-quality interactions weekly |
| 7. spends half of the day unpredictable environment, can make few choices | 7. somewhat engaged with enrichment weekly | 7. the dog is socially isolated most days and has moderate-quality interactions in between |
| 8. spends most of their time in unpredictable environment, can make few choices | 8. poorly engaged with enrichment monthly | 8. the dog is socially isolated most days and has poor social interactions in between |
| 9. spends the majority of time in unpredictable environment, can make very few choices | 9. rarely engages with any forms of enrichment | 9. the dog is socially isolated for 50% of each day and has poor social interactions the rest of the time |
| 10. spends almost all of their time in highly unpredictable environment, cannot make any choices | 10. has no enrichment or does not engage with enrichment | 10. the dog is constantly socially isolated |

| **Procedural** |  |  |  |
| --- | --- | --- | --- |
| **Behaviour during assessment** | **Change in daily routine** | **Handling during assessment** | **Procedure pain** |
| 1. is calm and actively seeks interaction from assessor/s | 1. Procedure / disruption to day < 15 minutes | 1. displays minimal signs of stress when handled, is calm and tolerates being handled well | 1. no procedure required |
| 2. is mostly relaxed and shows mild signs of stress to few triggering events | 2. Procedure / disruption to day < 30 minutes | 2. minimal movement when handled, sometimes licks lips, yawns or shows other appeasement behaviour | 2. minor procedure with no expected pain |
| 3. is somewhat relaxed and shows mild signs of stress to some triggering events | 3. Procedure / disruption to day 30 mins - 1 hour | 3. minimal movement when handled, licks lips, yawns, or shows appeasement behaviour frequently | 3. minor procedure longer duration with no expected pain |
| 4. is not relaxed and shows moderate signs of stress to few triggering events | 4. Procedure / disruption to day 1-2 hours | 4. some slow movement when handled, turns head away from handler, slow panting, displays more than two signs of stress such as ears back and tail down | 4. minor procedure with short mild pain |
| 5. shows moderate signs of stress to some triggering events | 5. Procedure / disruption to day 3-4 hours | 5. moderate movement when handled, fast panting, displays more than two signs of stress such as ears back, tail tucked and furrowed brow | 5. moderate procedure with short duration of transient pain |
| 6. shows moderate signs of stress to all triggering events | 6. Procedure / disruption to day >4 hours | 6. some attempt to escape, fast movements, tense body and tense closed mouth | 6. moderate procedure, longer in duration with transient pain |
| 7. shows major signs of stress to few triggering events | 7. Procedure / disruption to day >6 hours | 7. moderate attempts to escape, fast movements or frozen and staring, tense and trembling | 7. moderate/severe procedure, with pain lasting >12 hours |
| 8. shows major signs of stress to some triggering events | 8. Procedure / disruption to day >8 hours | 8. strong attempts to escape when handled or frozen, lifts lips and shows teeth | 8. severe procedure with pain lasting >24 hours |
| 9. shows major signs of stress to all triggering events | 9. Procedure / disruption to day >12 hours | 9. will violently attempt to escape when handled or frozen, growls and barks | 9. severe procedure with pain or complications lasting > 48 hours |
| 10. cannot cope being in the environment, is extremely shut-down or aggressive and shows major signs of stress | 10. Procedure / disruption to day >24 hours | 10. cannot be handled, growls and attempts to bite when approached | 10. extensive procedure resulting in severe long-term pain or complications |
